# Progression to end-stage renal disease is reduced with eculizumab in patients with atypical haemolytic uraemic syndrome

**DOI:** 10.1186/cc14409

**Published:** 2015-03-16

**Authors:** J Vande Walle, S Johnson, E Harvey, J Kincaid

**Affiliations:** 1 University Hospital Ghent, Belgium; 2 Medicus Economics, LLC, Boston, MA, USA; 3 Alexion Pharmaceuticals, Cheshire, CT, USA

## Introduction

Atypical haemolytic uraemic syndrome (aHUS) is associated with severe kidney damage; almost one-half of adults with aHUS progress to end-stage renal disease (ESRD) after the first episode [1]. Two prospective clinical trials have assessed the efficacy of eculizumab (Ecu) in patients with aHUS [2]. We now evaluate data on progression to ESRD before and during Ecu treatment.

## Methods

Patients with chronic kidney disease (CKD) stage 1 to 4 were analysed for progression to an ESRD event (two consecutive glomerular filtration rate measurements <15 ml/minute/1.73m^2^ (CKD stage 5)). ESRD incidence rate ratios during supportive care (SC) and Ecu treatment phases were calculated using a negative binomial regression analysis. Kaplan-Meier analyses were calculated for all patients and stratified by CKD stages 2 to 4 at baseline. Hazard ratios (HR) were calculated from Cox proportional hazard models.

## Results

**Figure 1:**
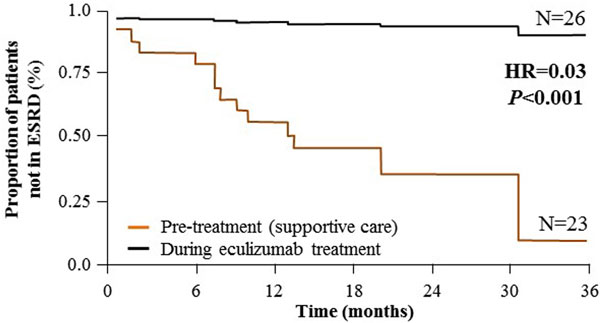
**Kaplan-Meier analysis of ESRD progression for SC and Ecu treatment for all patients**.

The SC and Ecu treatment phases included 32 and 33 patients, respectively. With SC, during a median (range) of 211 (7 to 745) days, 13 (41%) patients had a total of 16 ESRD events. On Ecu treatment, during a median (range) of 924 (73 to 1,254) days, three (9%) patients had a total of five ESRD events. The ESRD event rate was 92% lower during Ecu treatment versus the SC phase (0.36 vs. 0.07; *P *= 0.001); the incidence rate ratio was 0.08 (95% CI = 0.02 to 0.37; *P *= 0.001). HR for progression to ESRD for patients on Ecu versus SC was 0.03 (95% CI <0.01 to 0.34), a 97% reduction (Figure 1). Stratification by baseline CKD stage showed no patients with CKD stage 2 or 3 at baseline progressed to ESRD over 3 years of Ecu treatment.

## Conclusion

Ecu treatment reduces the number of ESRD events and the rate of progression to ESRD; thus initiation of Ecu early after aHUS diagnosis may prevent cumulative kidney damage and progression to ESRD.
